# Tolerance to nascent protein misfolding stress requires fine-tuning of the cAMP/PKA pathway

**DOI:** 10.1016/j.jbc.2021.100690

**Published:** 2021-04-22

**Authors:** Paraskevi Kritsiligkou, Karol Nowicki-Osuch, Zorana Carter, Chris J. Kershaw, Declan R. Creamer, Alan J. Weids, Chris M. Grant

**Affiliations:** Faculty of Biology, Medicine and Health, The University of Manchester, Manchester, UK

**Keywords:** stress response, protein kinase A (PKA), cell signaling, peroxiredoxin, oxidation–reduction (redox), gene expression, hydrogen peroxide, oxidative stress, protein misfolding, yeast, AZC, azetidine-2-carboxylic acid, CBD, cAMP-binding domain, ESR, environmental stress response, Hsf1, heat shock factor 1, mPEG-MAL, methoxypolyethylene glycol maleimide, PKA, protein kinase A, Prx, peroxiredoxin, ROS, reactive oxygen species

## Abstract

Protein aggregation is the abnormal association of misfolded proteins into larger, often insoluble structures that can be toxic during aging and in protein aggregation-associated diseases. Previous research has established a role for the cytosolic Tsa1 peroxiredoxin in responding to protein misfolding stress. Tsa1 is also known to downregulate the cAMP/protein kinase A (PKA) pathway as part of the response to hydrogen peroxide stress. However, whether the cAMP/PKA pathway is involved in protein misfolding stress is not known. Using transcriptomics, we examined the response to protein misfolding stress and found upregulation of numerous stress gene functions and downregulation of many genes related to protein synthesis and other growth-related processes consistent with the well-characterized environmental stress response. The scope of the transcriptional response is largely similar in wild-type and *tsa1* mutant strains, but the magnitude is dampened in the strain lacking Tsa1. We identified a direct protein interaction between Tsa1 and the Bcy1 regulatory subunit of PKA that is present under normal growth conditions and explains the observed differences in gene expression profiles. This interaction is increased in a redox-dependent manner in response to nascent protein misfolding, *via* Tsa1-mediated oxidation of Bcy1. Oxidation of Bcy1 causes a reduction in cAMP binding by Bcy1, which dampens PKA pathway activity, leading to a targeted reprogramming of gene expression. Redox regulation of the regulatory subunit of PKA provides a mechanism to mitigate the toxic consequences of protein misfolding stress that is distinct to stress caused by exogenous sources of reactive oxygen species.

Even though conformational flexibility is required for protein function, forming abnormal conformations can result in protein misfolding and aggregation (1). Protein aggregation is defined as the abnormal association of misfolded proteins into larger, often insoluble structures (2). It can be caused by exposing hydrophobic residues that would otherwise be buried within native protein folds and therefore protected from aberrant protein interactions (1, 3). Newly synthesized proteins are particularly vulnerable to misfolding and aggregation due to translational errors during their synthesis, as well as the necessity to fold from linear peptide chains into their final native structures (4). To maintain protein homeostasis, cells contain an arsenal of molecular chaperones able to detect non-native, misfolded proteins and act upon them to prevent aggregation or to mitigate their toxic consequences (1, 5). Despite the presence of chaperones, the levels of cellular protein aggregation can be increased by genetic, cellular, and environmental factors, including advancing age (2, 4, 6). Protein aggregation is also increased in response to stress conditions such as exposure to high temperatures and oxidants, which result in unfolding of the native structures of proteins (7, 8).

There are now many established links between protein aggregate formation and exposure to reactive oxygen species (ROS) (7). Not surprisingly therefore, enzymes with antioxidant activity, such as peroxiredoxins (Prxs), have been extensively linked with protein aggregation. Work in bacteria has shown that the absence of a peroxiredoxin and the associated higher levels of ROS results in increased protein aggregation suggesting that the Prx normally protects against protein aggregate formation (9). Similarly, in the yeast *Saccharomyces cerevisiae*, the main cytosolic Prx, Tsa1, has been extensively linked with protein aggregation. Loss of Tsa1 results in higher levels of proteins within aggregate fractions, especially ribosomal proteins (10, 11, 12). Mutants lacking Tsa1 are also particularly sensitive to azetidine-2-carboxylic acid (AZC), a proline analogue used to induce protein aggregation, suggesting that Tsa1 is required for tolerance to nascent protein misfolding stress (13). The protein aggregates induced by protein misfolding form adjacent to mitochondria and result in ROS formation from active mitochondria (13). Additionally, Tsa1 can become hyperoxidized in response to hydrogen peroxide exposure, which triggers its formation into high-molecular-weight structures with chaperone activity (11). More recently, overoxidation of Tsa1 has been found to extend life span by recruiting chaperones to damaged and aggregated proteins formed during aging and in response to hydrogen peroxide stress (14). Thus, Tsa1 has multiple potential roles during protein aggregation stress functioning as an antioxidant and chaperone.

There is increasing awareness that in addition to causing oxidative stress, ROS play important roles as signaling molecules in the regulation of many biological processes (15, 16). This regulatory role for ROS is mediated by the oxidation of cysteine thiol groups, which are found in the catalytic or regulatory domains of enzymes. These so-called “thiol switches” can be reversibly oxidized and transiently regulate diverse biological processes such as signal transduction, wound healing, immune function, tumorigenesis, and aging (17). Cysteine residues react relatively slowly with hydrogen peroxide, but reactivity can be significantly enhanced by their deprotonation, which is dependent on the local protein environment. This means that some redox-regulated proteins are directly oxidized by ROS, although it is now known that most regulatory thiol oxidation is mediated by protein catalysts (18). Key to this regulation are thiol peroxidases including the Prx family of enzymes, which act as both hydrogen peroxide sensors and thiol oxidizers (19, 20). This generally involves redox relay reactions where an oxidized Prx oxidizes specific thiol residues in target proteins (21).

Previous studies have implicated Tsa1 in moderating the response to hydrogen peroxide stress *via* downregulation of the highly conserved cAMP/protein kinase A (PKA) pathway (22). More recently, Tsa1 was shown to suppress PKA activity by oxidizing a conserved Cys residue in the Tpk1 catalytic subunit of PKA (23). In this current study, we investigated how Tsa1 mediates tolerance to protein misfolding and aggregation stress by studying the transcriptomic alterations that occur in response to AZC exposure. We show that downregulation of the PKA pathway promotes tolerance to nascent protein misfolding stress and the importance of Tsa1 lies in its signalling response to proteotoxic stress. We used a proteomic screen to identify factors that interact with Tsa1 following AZC stress and identified a direct interaction with the Bcy1 regulatory subunit of PKA that is increased upon protein misfolding stress in a redox-dependent manner. Tsa1-mediated oxidation of Bcy1 causes a reduction in cAMP binding by Bcy1, which dampens PKA pathway activity leading to a targeted reprogramming of gene expression that promotes tolerance to protein misfolding and aggregation. The Tsa1-Bcy1 interaction was not affected by the addition of exogenous hydrogen peroxide and no increase in Bcy1 oxidation was observed in response to hydrogen peroxide exposure. Redox regulation of the regulatory subunit of PKA therefore provides a mechanism to mitigate the toxic consequences of protein misfolding stress that is distinct to the regulation of the catalytic subunit of PKA caused by the addition of exogenous hydrogen peroxide.

## Results

### Protein misfolding stress causes large-scale transcriptional changes

As Prxs have functions in antioxidant defense, as chaperones, and in redox signaling, it is unclear which activity is required following nascent protein misfolding stress. We therefore used RNA sequencing (RNA-Seq) to examine how *tsa1* mutant cells respond at the transcriptional level to an increase in protein misfolding. AZC is competitively incorporated into proteins in place of proline, where it alters the conformation of the polypeptide backbone resulting in decreased thermal stability and nascent protein misfolding and aggregation (24). In yeast, the transcriptional response to proteotoxic stress is primarily mediated by heat shock factor 1 (Hsf1) and the Msn2/4 transcription factors. Hsf1 regulates chaperones and other factors involved in protein folding and misfolding and aggregate clearance that would be required during proteostasis stress (25). The scope of the Msn2/4 regulon is much broader than the Hsf1 regulon and is commonly referred to as the environmental stress response (ESR) (26, 27). A previous microarray-based study showed that AZC stress induces the expression of Hsf1 regulated genes, without activating the ESR (28). This study exposed cells to high concentrations of AZC (50 mM) for 5 h and we wanted to examine the response to conditions that we have established cause protein misfolding and aggregation (5 mM AZC for 2 h) without significantly decreasing cell viability (13).

**Figure 1:**
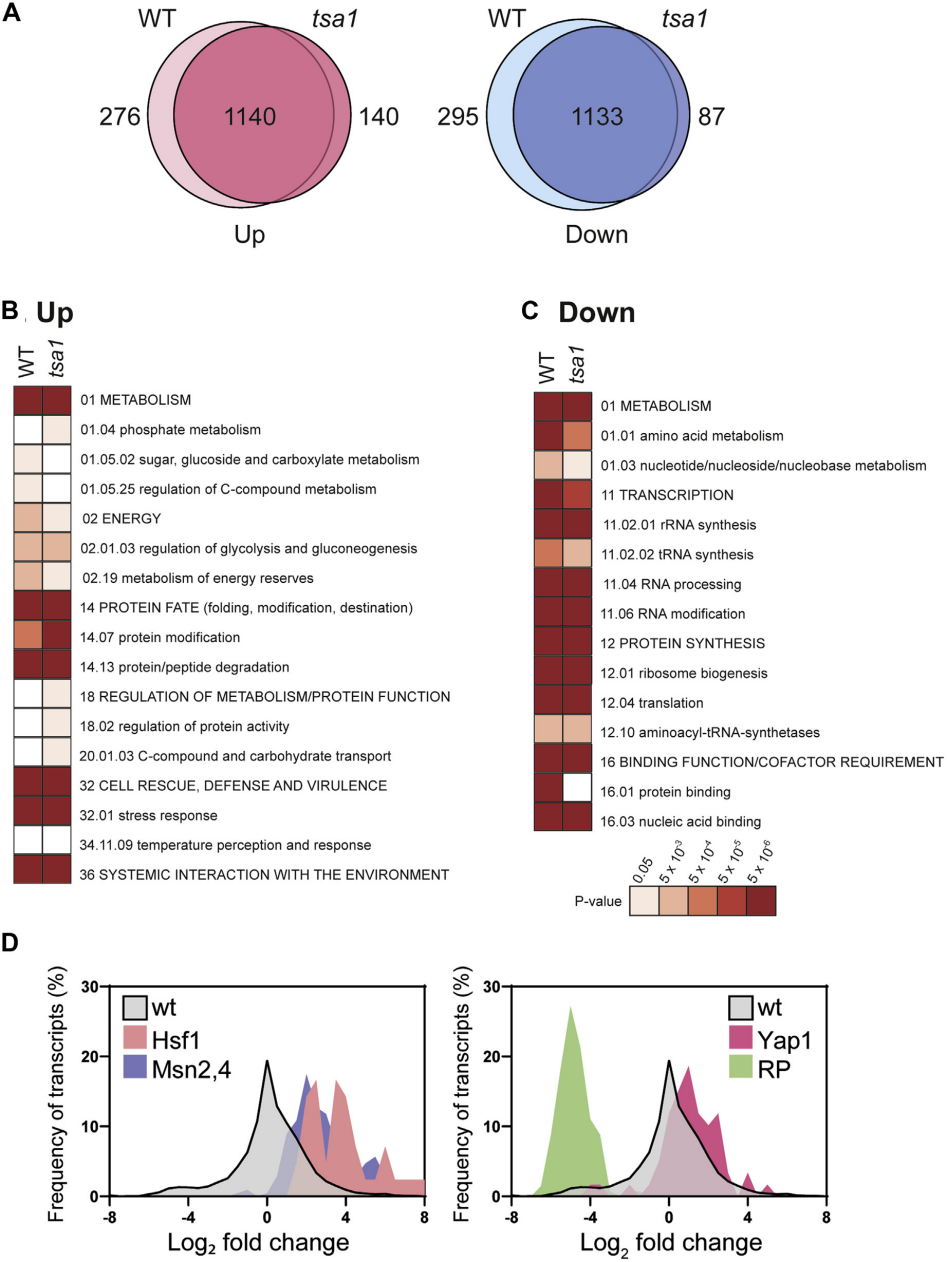
**Protein misfolding stress causes large-scale changes in gene expression.***A*, the scope of the transcriptional response to AZC stress is similar in wild-type and *tsa1* mutant strains. Wild-type and *tsa1* mutant strains were exposed to 5 mM AZC for 2 h and changes in transcript abundance determined using RNA-Seq. Euler diagrams show the overlap in transcripts that change by greater than twofold in the wild-type and *tsa1* mutant strains. Similar transcripts are upregulated (*B*) or downregulated (*C*) in response to AZC stress in wild-type and *tsa1* mutant strains. For each condition, significantly enriched functional categories within the datasets were determined (FDR < 5%) and results are ordered on MIPS category classification. Confidence of each classification category is shown as Bonferroni corrected *p*-values. *D*, Hsf1 and Msn2,4 target genes are upregulated and ribosomal protein genes are downregulated in response to protein misfolding stress. Transcriptional changes in abundance (log_2_ fold change) for the whole transcriptome (*gray*) are shown compared with transcriptional changes for Hsf1 targets (*pink*), Msn2,4 targets (*blue*), Yap1 targets (*red*), and ribosomal proteins (*green*) in a wild-type strain.

Analysis of the transcriptome in a wild-type strain revealed that many transcripts are significantly altered by at least twofold following exposure to AZC including 1416 transcripts that are significantly increased and 1408 transcripts that are decreased in abundance compared with an untreated culture (Fig. 1*A*). This is a much larger response than that observed for higher AZC concentrations, where, for example, 217 transcripts were reported to be induced by greater than threefold (28) compared with 873 transcripts that showed a similar fold change in response to the lower AZC treatment.

To better understand the transcriptional response to AZC stress, we searched for significantly enriched (5% FDR) functional categories within our datasets using the MIPS Functional Catalogue (29). In contrast to the treatment with a higher concentration of AZC (28), we found that many of the induced genes are expected as part of the ESR (Fig. 1, *B* and *C*). This included a number of major categories that are commonly altered during diverse stress conditions including oxidative stress, heat shock, and starvation responses (26). AZC stress resulted in the induction of genes affecting numerous protein folding, modification, and degradation factors, which would be required during conditions that promote protein misfolding (Fig. 1*B*). There was also an induction of genes involved in energy metabolism and alternative carbon source utilization, which might arise due to decreases in cellular ATP concentrations as a consequence of chaperone-dependent protein folding activity (26). AZC stress caused the downregulation of gene expression for many genes related to protein synthesis (rRNA synthesis and processing, tRNA synthesis and processing, ribosome biogenesis, translation) presumably reflecting the need to conserve energy and to reduce the likelihood of further aberrant protein formation (30). Similarly, other growth-related processes including transcription, nucleotide, amino acid, and other metabolic processes were downregulated in response to AZC stress (Fig. 1*C*).

Analysis of the transcriptional response to AZC stress revealed that the induced genes include known targets of Hsf1 (Fig. 1*D*), similar to the previous analysis of the transcriptional response to higher AZC concentrations (28). In contrast to this previous study, however, the induced genes also included targets of the Msn2/4 transcription factors (Fig. 1*D*). Despite the finding that protein misfolding stress induces ROS formation (13), a less striking activation of Yap1 target genes was observed in response to AZC stress (Fig. 1*D*). This correlates with the finding that *yap1* mutants do not show increased sensitivity to AZC stress (13). Furthermore, a comparison of the response to AZC stress *versus* heat stress (Fig. S1*A*) or oxidative stress (Fig. S1*B*) revealed a modest similarity between AZC and heat stress (R^2^ = 0.50), whereas the similarity between AZC and oxidative stress was less strong (R^2^ = 0.33). Many stress conditions inhibit the expression of ribosomal protein and assembly genes (RiBi regulon) through TOR and RAS/PKA-pathway dependent signaling (31, 32, 33, 34) and analysis of 136 ribosomal protein transcripts revealed that they were strongly downregulated (Fig. 1*D*). Taken together, these data indicate that AZC stress induces the upregulation of the heat shock response (Hsf1 targets) and the ESR (Msn2/4 targets) and downregulation of ribosomal biosynthesis.

### The transcriptional response to protein misfolding stress is dampened in a *tsa1* mutant

We next examined the transcriptional response to AZC in a *tsa1* mutant to determine whether Tsa1 is required for the response to protein misfolding stress at the gene expression level. Under nonstress conditions, relatively modest numbers of transcripts are altered in abundance in a *tsa1* mutant compared with a wild-type strain (Fig. S2*A*). This included 170 and 34 transcripts that were up- or downregulated by greater than twofold, respectively. Functional analysis of these transcripts revealed that the upregulated transcripts in a *tsa1* mutant were enriched for functions related to energy and stress responses (Fig. S2*B*). Upregulation of stress response genes, especially those involved in the oxidative stress response, might have been expected in a *tsa1* mutant, as Tsa1 is one of the main cellular antioxidants.

The expression of many transcripts is altered in the *tsa1* mutant in response to AZC stress including 1280 and 1220 transcripts that are increased or decreased, respectively. There was a large overlap in the transcripts identified as altered in the wild-type and *tsa1* mutant strains (Fig. 1*A*). Accordingly, the same functional categories were predominantly affected by AZC stress in the *tsa1* mutant strain suggesting that the scope of the transcriptional response to AZC stress is largely similar in wild-type and *tsa1* mutant strains (Fig. 1, *B* and *C*).

**Figure 2:**
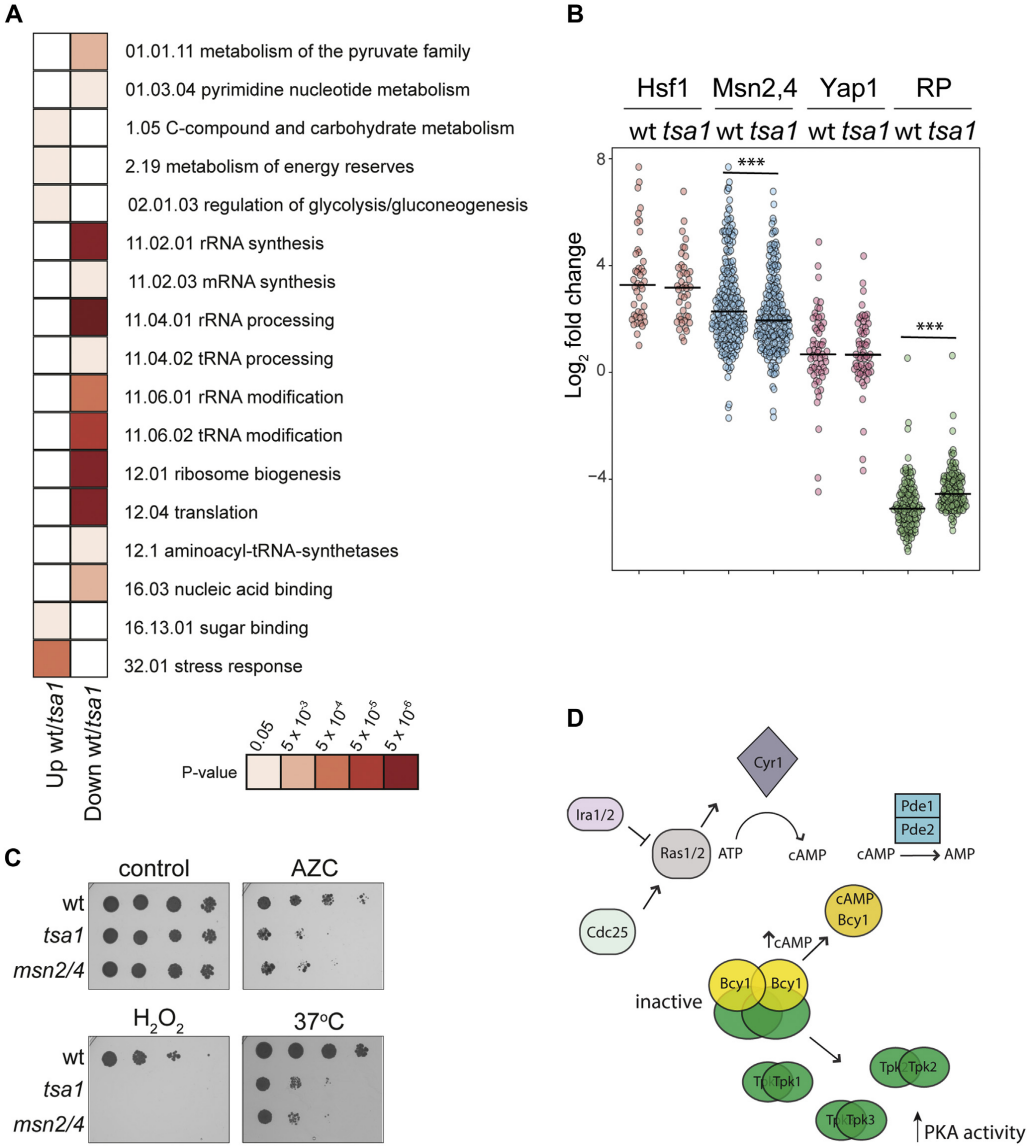
**The transcriptional response to protein misfolding stress is dampened in a *tsa1* mutant.***A*, 505 transcripts are not as upregulated in a *tsa1* mutant compared with a wild-type strain and 802 transcripts are not as downregulated. Significantly enriched functional categories within these transcripts were determined (FDR < 5%) and results are ordered on MIPS category classification numbers. Confidence of each classification category is shown as Bonferroni corrected *p*-values. *B*, Msn2,4 target mRNAs are not as upregulated and ribosomal protein transcripts are not as downregulated in a *tsa1* mutant compared with a wild-type strains following AZC stress. Transcriptional changes in abundance (log_2_ fold change) are shown for Hsf1-targets, Msn2,4-targets, Yap1-targets, and ribosomal proteins in wild-type and *tsa1* mutant strains; significance, ∗∗∗*p* < 0.001. *C*, mutant strains deleted for *MSN2* and *MSN4* are sensitive to AZC, hydrogen peroxide, and heat stress. Strains were grown to exponential phase and the A_600_ adjusted to 1, 0.1, 0.01, or 0.001 before spotting onto SD plates containing 0.3 mM AZC, 0.5 mM hydrogen peroxide, or incubation at 37 °C. *D*, simplified schematic of the cAMP/PKA pathway. Active Ras2 stimulates the yeast adenylate cyclase (Cyr1), which synthesizes cAMP from ATP. cAMP levels are further regulated by phosphodiesterase (Pde1/2). In the absence of cAMP, PKA is present as an inactive heterotetrameric form comprising two catalytic (encoded by *TPK1*-*3*) and two Bcy1 regulatory subunits. In response to increasing cAMP concentration, Bcy1 is bound by cAMP, resulting in its dissociation from the PKA complex and activation of TPK kinase activity.

We next assessed whether the magnitude of the transcriptional response to protein misfolding stress is similar in the wild-type and *tsa1* mutant strains. For this analysis, we compared the fold change in transcript levels in a wild-type with those in the *tsa1* mutant following AZC stress. This analysis identified that 505 transcripts were not as upregulated in the *tsa1* mutant compared with the wild-type strain and 802 transcripts were not as downregulated. The not as upregulated transcripts included transcripts encoding factors involved in metabolism (carbohydrate metabolism and energy) and stress responses, whereas many factors affecting translation and protein synthesis were not as downregulated in response to AZC stress in the *tsa1* mutant (Fig. 2*A*). *In silico* analysis searching for transcription factor binding motifs showed a strong enrichment for binding sites for Msn2/4 in the not as upregulated genes. In agreement with this finding, direct comparison of the fold change in transcript levels in wild-type and *tsa1* mutant strains confirmed that Msn2/4 targets were not as upregulated in response to AZC in a *tsa1* mutant compared with a wild-type strain (Fig. 2*B*). In contrast, similar upregulation of the Hsf1 and Yap1 regulons was observed in both strains following AZC exposure (Fig. 2*B*). Additionally, transcripts encoding ribosomal proteins were not as downregulated in response to AZC stress in a *tsa1* mutant compared with the wild-type strain (Fig. 2*B*). We further confirmed the role of Msn2/4 in the response to protein misfolding and aggregation stress as a mutant lacking these transcription factors showed sensitivity to AZC stress, comparable to the sensitivity of a *tsa1* mutant strain (Fig. 2*C*). The *msn2 msn4* mutant was also sensitive to heat and oxidative stresses as would be expected.

### Tsa1 interacts with Bcy1 in a redox-dependent manner

We questioned how Tsa1 might influence Msn2/4 activity and ribosomal protein biogenesis and hypothesized that it could do so by directly interacting with factors that affect their regulatory pathways. Previous studies have used a resolving cysteine mutant (Tsa1-Cys171S) to stabilize transient disulfide interactions with the Yap1 transcription factor (35) and pyruvate kinase, Pyk1 (36). Additionally, the resolving cysteine mutant has been used to identify five potential Tsa1 targets under nonstress conditions (Pyk1, Vde1, Ssb1/Ssb2, Yol057w, Frd1) (36). Since none of these potential Tsa1 substrate proteins seems likely to regulate Msn2/4 and ribosomal protein biogenesis, we carried out a proteomic screen to identify such factors following nascent protein misfolding stress. We utilized an N-terminal Myc-tagged version of Tsa1 with a mutation in its resolving cysteine residue (Tsa1-C171S) to trap and detect redox-dependent interactions. Yeast cells were left untreated or treated with AZC or hydrogen peroxide prior to immunoprecipitation and mass spectrometry used to identify co-immunoprecipitating proteins. These experiments identified a number of unique proteins that interacted with Tsa1 in repeat experiments under each condition (Table 1). Most interestingly, Bcy1, the regulatory subunit of PKA, was found to interact with Tsa1 under AZC stress conditions.

**Table 1:** Proteins that co-immunoprecipitate with Tsa1-C171S

Unstressed	Hydrogen peroxide	AZC
Sub2	Rfa1	Bcy1
	Mlp2	Lys21
	Tor1	Hxk1
	Bud2	Srp1
	YMR102C	Zta1
	Ser3	Dak1
		Ede1
		Ser3

The cAMP/PKA pathway is a highly conserved glucose sensing and signaling pathway that plays a major role in the control of stress responses, metabolism, and cell proliferation (37). PKA is a heterotetrameric complex comprising two catalytic and two Bcy1 regulatory subunits (Fig. 2*D*). In *S. cerevisiae* there are three PKA catalytic subunit isoforms, encoded by three different genes, *TPK1-3*. Binding of cAMP to Bcy1 causes its dissociation from PKA and activation of the kinase activity of Tpk1-3 (Fig. 2*D*). The cAMP/PKA pathway regulates the ESR and ribosomal biosynthesis and hence, Bcy1 is an ideal candidate for regulating these stress responses following protein misfolding stress (38, 39).

**Figure 3:**
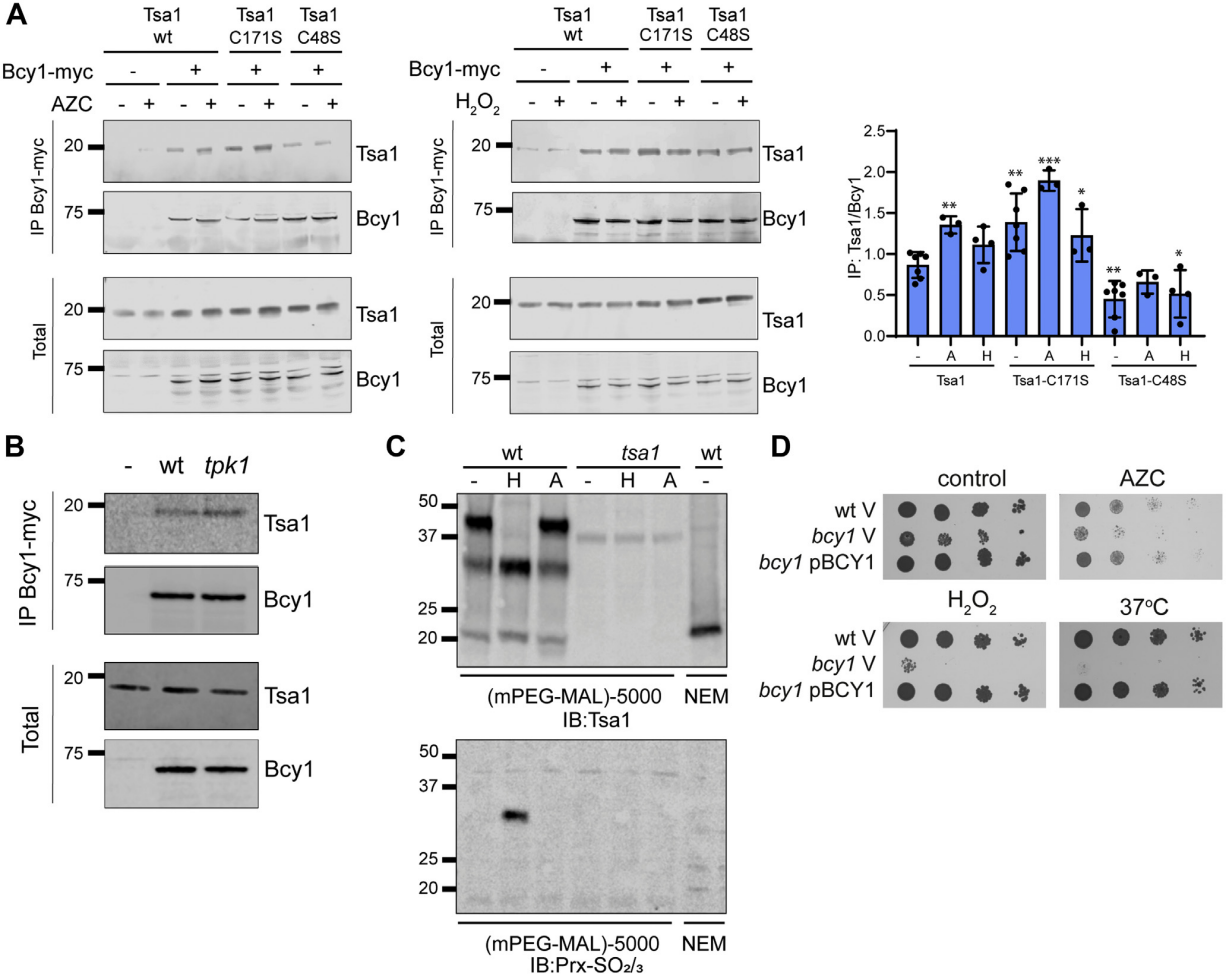
**Tsa1 interacts with the Bcy1 regulatory subunit of PKA.***A*, the interaction between Tsa1 and Bcy1 increases in a redox-dependent manner in response to AZC stress. Bcy1-Myc was immunoprecipitated from cells expressing wild-type Tsa1, a resolving cysteine *tsa1* mutant (C171S) or a catalytic cysteine *tsa1* mutant (C48S) during nonstress conditions and following AZC (5 mM) or hydrogen peroxide (0.4 mM) stress conditions for 2 h. Co-immunoprecipitation of Tsa1 was detected using an anti-Tsa1 antibody. Quantification is shown expressed as the ratio of Tsa1 co-immunoprecipitated by Bcy1. Data shown are the means from 3 to 7 independent biological repeat experiments ±S.D. Significance is indicated relative to the untreated wild-type strain (∗*p* < 0.05, ∗∗*p* < 0.01, ∗∗∗*p* < 0.001). *B*, loss of Tpk1 does not affect the co-immunoprecipitation of Bcy1 and Tsa1. *C*, the redox state of Tsa1 was assessed during nonstress conditions and following AZC (A) or hydrogen peroxide (H) stress conditions with the use of mPEG-Mal. SDS-PAGE gels were run under reducing conditions and immunoblots probed with antibodies specific for Tsa1 (*top panel*) or Prx-SO_2/3_ (*bottom panel*). *D*, mutants deleted for *BCY1* are sensitive to protein misfolding stress. Results are shown for the wild-type and *bcy1* mutant containing vector alone (V) and the *bcy1* mutant complemented with a plasmid expressing *BCY1*. Strains were grown to exponential phase and the A_600_ adjusted to 1, 0.1, 0.01, or 0.001 before spotting onto SD plates containing 0.3 mM AZC, 0.6 mM hydrogen peroxide, or incubation at 37 °C.

The interaction between Tsa1 and Bcy1 was verified using Myc-tagged Bcy1 in the reverse of the immunoprecipitation experiments used to identify Bcy1. In these experiments, plasmids expressing untagged wild-type or cysteine mutant versions of Tsa1 were used to complement a *tsa1* mutant strain. Tsa1 was co-immunoprecipitated with Bcy1-myc during normal growth conditions and this protein interaction increased following AZC stress (Fig. 3*A*). The increased interaction between Tsa1 and Bcy1 observed in response to AZC stress is redox-dependent, as it was stabilized in a resolving cysteine residue mutant (Tsa1-C171S) and decreased in a peroxidatic cysteine residue mutant (Tsa1-C48S) (Fig. 3*A*). The interaction between Tsa1 and Bcy1 was not as significantly increased in response to oxidative stress caused by the addition of exogenous hydrogen peroxide (Fig. 3*A*, right panel). A physical interaction between Tsa1 and the Tpk1 catalytic subunit of PKA has previously been reported, although there was no evidence for a Tsa1-Tpk1 disulfide bonded intermediate (23). We therefore tested whether Tpk1 is required for the Tsa1-Bcy1 interaction. However, loss of Tpk1 did not affect the co-immunoprecipitation of Tsa1 with Bcy1 confirming that there is no requirement for Tpk1 to mediate this interaction (Fig. 3*B*). It should be noted that this strain still expresses Tpk2 and Tpk3 and that they are partially functionally redundant with Tpk1, although it is not known if Tsa1 can interact with Tpk2 or Tpk3.

To better understand the differences between oxidative stress caused by protein misfolding and the addition of a bolus of hydrogen peroxide, we examined how the redox state of Tsa1 is affected. The *in vivo* redox state of Tsa1 was visualized by following the reactivity of its free sulfydryl groups with methoxypolyethylene glycol maleimide (mPEG-MAL). In this assay, cell extracts are made in the presence of trichloroacetic acid to prevent any thiol–disulfide interchange and mPEG-MAL used to alkylate cysteine residues in a free-SH but not in an oxidized state. Reaction with mPEG-MAL increases the molecular weight of Tsa1, which is observed as a mobility shift using SDS-PAGE. Tsa1 contains two cysteine residues and under normal conditions, Tsa1 was predominantly present in its fully reduced form (Fig. 3*C*). An intermediate band was also present corresponding to Tsa1 with one reduced and one oxidized thiol group. Oxidative stress caused by hydrogen peroxide addition resulted in loss of the fully reduced Tsa1 and accumulation of the partially oxidized form. In contrast, protein misfolding stress caused by AZC did not affect the redox state of Tsa1 as measured using mPEG-MAL. Probing the blots with antibodies specific for Prx-SO_2/3_ revealed that overoxidation of Tsa1 could be detected following hydrogen peroxide stress, but not following nascent protein misfolding stress (Fig. 3*C*, lower panel). These results show that hydrogen peroxide and protein misfolding stress do not affect Tsa1 in the same way and the overoxidation of Tsa1 seen in response to hydrogen peroxide might account for the less significant increase in the Tsa1-Bcy1 interaction following this oxidative stress.

Our immunoprecipitation data indicated a direct interaction between Tsa1 and the Bcy1 regulatory subunit of PKA that we reasoned could modulate PKA activity in response to protein misfolding stress. We therefore questioned whether mutants deleted for *BCY1* are sensitive to AZC stress. This was indeed the case and the sensitivity of a *bcy1* mutant to AZC could be complemented by reintroducing *BCY1* expressed from a plasmid, confirming that Bcy1 is required for the response to protein misfolding stress (Fig. 3*D*). The *bcy1* mutant was also sensitive to other stress conditions including heat and hydrogen peroxide stresses (Fig. 3*D*).

### Downregulation of the cAMP/PKA pathway promotes tolerance to nascent protein misfolding stress

**Figure 4:**
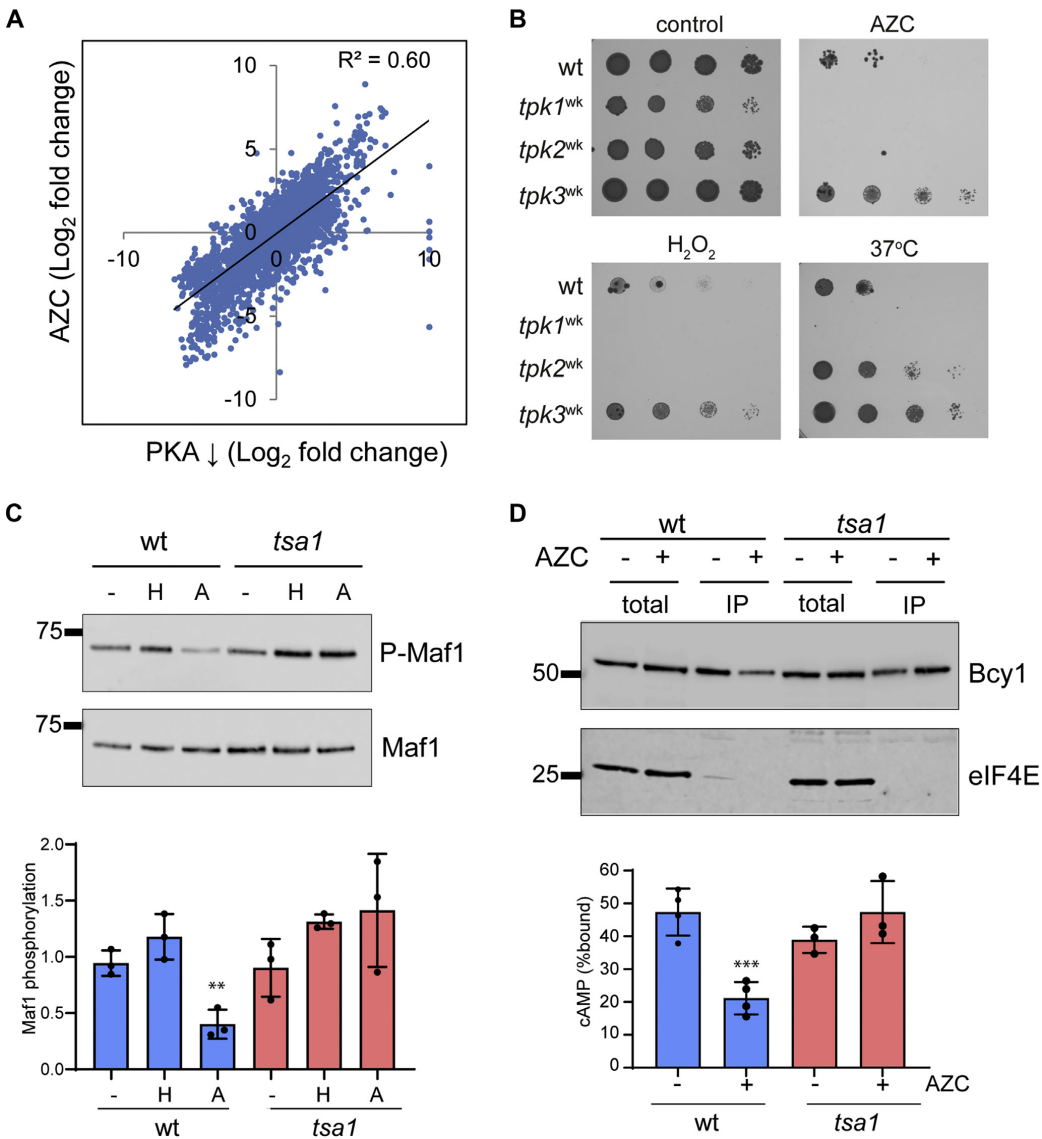
**Downregulation of the cAMP/PKA pathway promotes tolerance to nascent protein misfolding stress.***A*, the transcriptional response to AZC stress shows strong resemblance to the transcriptional response to PKA inhibition. A scatterplot is shown comparing the transcriptional response to AZC with that for chemical inhibition of PKA activity (25). *B*, the indicated mutants lack two of the Tpk isoforms and contain an attenuated mutant form of the remaining Tpk isoform with greatly reduced catalytic activity. Strains were spotted onto SD plates containing 0.15 mM AZC, 1.5 mM hydrogen peroxide, or incubation at 37 °C. *C*, the PKA phosphorylation status of Maf1 was assessed as a measure of PKA activity. Maf1 was immunoprecipitated from wild-type and *tsa1* mutant strains during nonstress conditions and following hydrogen peroxide (0.4 mM) or AZC (5 mM) stress conditions for 2 h. Phosphorylation of Maf1 (P-Maf1) was detected using an antibody that recognizes phosphorylated PKA substrates (RRXS∗/T∗ motif). Quantification is shown expressed as the ratio of Maf1 phosphorylation to total Maf1 protein. Data shown are the means from three independent biological repeat experiments ±S.D. Significance is indicated relative to the untreated wild-type or *tsa1* mutant strains (∗∗*p* < 0.01). *D*, cAMP-affinity chromatography. Whole-cell extracts (lanes 1–2 and 5–6) prepared from wild-type and *tsa1* mutant strains following control or AZC stress conditions were incubated with cAMP-agarose and bound proteins analyzed by immunoblot (lanes 3–4 and 5–6). Blots were probed with antibodies specific for Bcy1 (αFLAG) or eIF4E as a loading control. Quantification is shown as the means from four independent biological repeat experiments ±S.D., ∗∗*p* < 0.01.

To further examine the role of the cAMP/PKA pathway, we compared the transcriptional response to AZC stress with the response to PKA inhibition. In agreement with our finding that AZC-induced genes include Msn2/4-target genes and repressed genes include ribosomal protein genes, there was good resemblance (R^2^ = 0.60) between the AZC stress response (our analysis) and the transcriptional response to chemical inhibition of PKA activity using a previously published dataset (25) (Fig. 4*A*).

To confirm the role of PKA in protein misfolding stress, we examined whether genetically ablating PKA activity promotes AZC tolerance. No single Tpk isoform is essential for viability, but a triple mutant is nonviable and at least one isoform is required for viability (40). We therefore used attenuated mutants that lack two of the Tpk isoforms and contain a mutant form of the remaining Tpk isoform with greatly reduced catalytic activity (referred to as weak *tpk* mutant strains) (41, 42). We observed that the strains with weak Tpk1 or Tpk2 activity were more sensitive to AZC stress, whereas the weak *tpk3* mutant was more resistant than a wild-type strain (Fig. 4*B*). The weak *tpk3* mutant was also more resistant to hydrogen peroxide stress and heat stress suggesting that it is a multistress-resistant mutant strain. The stress resistance of the weak *tpk3* probably does not arise due to any Tpk-isoform specificity as the residual Tpk activity in the weak *tpk3* mutant is thought to be higher than that in the weak *tpk1* and *tpk2* mutants and, for example, growth defects including slow growth are more severe in the weak *tpk1* and *tpk2* mutants than in the weak *tpk3* mutant (41, 42, 43), which might explain the apparent stress sensitivity in these mutants. These data also suggest that while suppression of PKA activity is needed in response to protein misfolding stress, some residual PKA activity is nevertheless required. Therefore, there may be an optimal level of PKA activity that is required for tolerance to this type of stress.

To confirm that the PKA pathway is downregulated upon AZC stress, we assessed the PKA phosphorylation status of a known PKA target. Maf1 is a highly conserved regulator of RNA polymerase III that is negatively regulated by PKA phosphorylation (44). Maf1 was immunoprecipitated from wild-type and *tsa1* mutant strains and its phosphorylation status was assessed using an antibody that recognizes phosphorylated PKA substrates. This antibody was raised against phosphorylated-PKA substrates (RRXS∗/T∗ motif) and has previously been used to assess PKA activity in cells (40, 41, 42). In agreement with previous observations (44), Maf1 was phosphorylated during nonstressed conditions (Fig. 4*C*). This phosphorylation was significantly decreased in a Tsa1-dependent manner in response to AZC stress. Further emphasizing the differences between the oxidative stress caused by nascent protein misfolding stress and exogenous hydrogen peroxide addition, PKA phosphorylation of Maf1 was not decreased in response to hydrogen peroxide stress (Fig. 4*C*).

We next questioned how protein misfolding stress might promote suppression of PKA activity *via* its Bcy1 interaction. The kinase activity of the PKA holoenzyme is normally inhibited by the Bcy1 regulatory subunit, but cAMP binding to Bcy1 promotes activation of the catalytic subunits (37). We therefore measured cAMP binding by Bcy1 using cAMP-agarose purification to test whether protein misfolding stress might affect Bcy1 cAMP binding as a mechanism to decrease PKA activity. We found that treating cells with AZC reduced Bcy1 cAMP binding by more than twofold, dependent on the presence of Tsa1 (Fig. 4*D*). This Tsa1-dependent reduction in cAMP binding would decrease PKA activity, promoting tolerance to protein misfolding stress.

### Bcy1 is oxidized in response to protein misfolding stress

**Figure 5:**
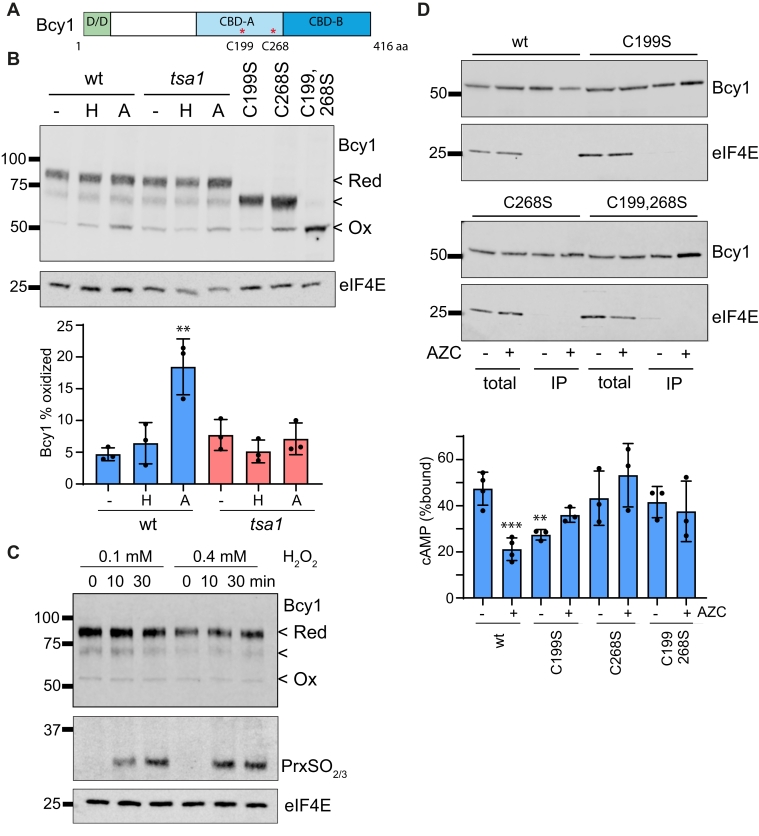
**Redox control of Bcy1 in response to protein misfolding stress.***A*, schematic of Bcy1. R-subunits contain an N-terminal region responsible for dimerization and docking (D/D) and two cAMP-binding domains (CBD) located at the C-terminus, which each bind two molecules of cAMP. Bcy1 contains two cysteine residues (Cys199, Cys268) both of which are located in CBD-A. *B*, Bcy1 is oxidized in response to AZC stress dependent on the presence of Tsa1. Whole-cell extracts were prepared from cells grown under nonstress and hydrogen peroxide (0.4 mM) or AZC (5 mM) stress conditions for 2 h. Proteins were precipitated with TCA and free thiols modified by reaction with mPEG-MAL. Blots were probed with antibodies specific for Bcy1 (αFLAG). Fully oxidized (Ox), partially oxidized (<), and fully reduced (*Red*) forms of Bcy1 are indicated. Quantification is shown for the fully oxidized form expressed as a percentage of fully reduced and fully oxidized Bcy1. Data shown are the means of three independent biological repeat experiments ±S.D., ∗∗*p* < 0.01. Blots were probed with antibodies specific for eIF4E as a loading control. *C*, Bcy1 oxidation was not observed in response to lower concentrations of hydrogen peroxide or earlier time points. Whole-cell extracts were prepared from cells grown under nonstress and following exposure to hydrogen peroxide (0.1, 0.4 mM) for 10 or 30 min. Proteins were precipitated with TCA and free thiols modified by reaction with mPEG-MAL. Blots were probed with antibodies specific for Bcy1 (αFLAG), Prx-SO_2/3_, and eIF4E. *D*, cAMP-affinity chromatography shows that the decrease in Bcy1 cAMP binding requires Bcy1 cysteine residues. Whole-cell extracts from the indicated strains (lanes 1–4) were incubated with cAMP-agarose and bound proteins analyzed by immunoblot (lanes 5–8). Blots were probed with antibodies specific for Bcy1 (αFLAG) or eIF4E as a loading control. Quantification is shown as the means from 3 to 4 independent biological repeat experiments ±S.D., ∗∗*p* < 0.01.

Since we found that the interaction between Tsa1 and Bcy1 is increased in a redox-dependent manner, we next examined the requirement for Bcy1 cysteine residues in the response to AZC stress. PKA regulatory subunits typically contain two cAMP-binding domains (CBD) located at their C-terminus, which each bind two molecules of cAMP. The yeast regulatory subunit Bcy1 contains two cysteine residues (Cys199, Cys268) that are both located within CBD-A (Fig. 5*A*).

We examined whether the oxidation state of Bcy1 cysteine residues is altered in response to AZC stress using mPEG-MAL. Under normal growth conditions, Bcy1 was predominantly present in its fully reduced form, as the migration of most of the protein was shifted to a higher molecular weight compared with a mutant lacking both cysteine residues (Fig. 5*B*). Loss of a single cysteine residue (C199S or C268S) resulted in an intermediate mobility shift. Comparison with the cysteine mutants suggested that Bcy1 with individual or both cysteine residues oxidized is present at relatively low levels during normal growth conditions (Fig. 5*B*). However, treatment with AZC increased the proportion of fully oxidized Bcy1 by approximately fourfold (Fig. 5*B*). The increased oxidation of Bcy1 was abrogated in a *tsa1* mutant indicating that Tsa1 is required to promote the oxidation of Bcy1 in response to AZC stress.

In contrast to AZC, no significant increase in Bcy1 oxidation was observed in response to hydrogen peroxide stress (Fig. 5*B*). It is perhaps surprising that protein misfolding stress but not exposure to exogenous hydrogen peroxide bolus promotes Bcy1 oxidation, given that we have previously shown that AZC stress results in mitochondrial ROS generation (13). These experiments were performed by exposing cells to 0.4 mM hydrogen peroxide for 2 h and we tested whether using lower hydrogen peroxide concentrations or shorter exposure times might promote Bcy1 oxidation. However, treatments with 0.1 mM or 0. 4 mM hydrogen peroxide for 10 or 30 min did not affect the oxidation state of Bcy1 (Fig. 5*C*). We further examined the redox state of Tsa1 with antibodies specific for Prx-SO_2/3_ and found that these milder hydrogen peroxide stress conditions resulted in overoxidation of Tsa1 (Fig. 5*C*), further confirming that overoxidation of Tsa1 is detected following hydrogen peroxide stress, but not following nascent protein misfolding stress.

We used cAMP-agarose purification to determine whether Bcy1 cysteine residues are required for the decrease in cAMP binding observed in response to protein misfolding stress. cAMP binding by the single C268S or double C199,268S mutant versions of Bcy1 was unaffected during normal nonstress growth conditions (Fig. 5*D*). In contrast, cAMP binding was reduced in the C199S Bcy1 mutant compared with wild-type Bcy1, suggesting that loss of Cys199 alone is sufficient to disrupt cAMP binding by Bcy1. Additionally, no decrease in cAMP binding was observed in response to AZC stress for any of the cysteine mutants confirming that redox regulation of Bcy1 is required to moderate cAMP binding (Fig. 5*D*).

**Figure 6:**
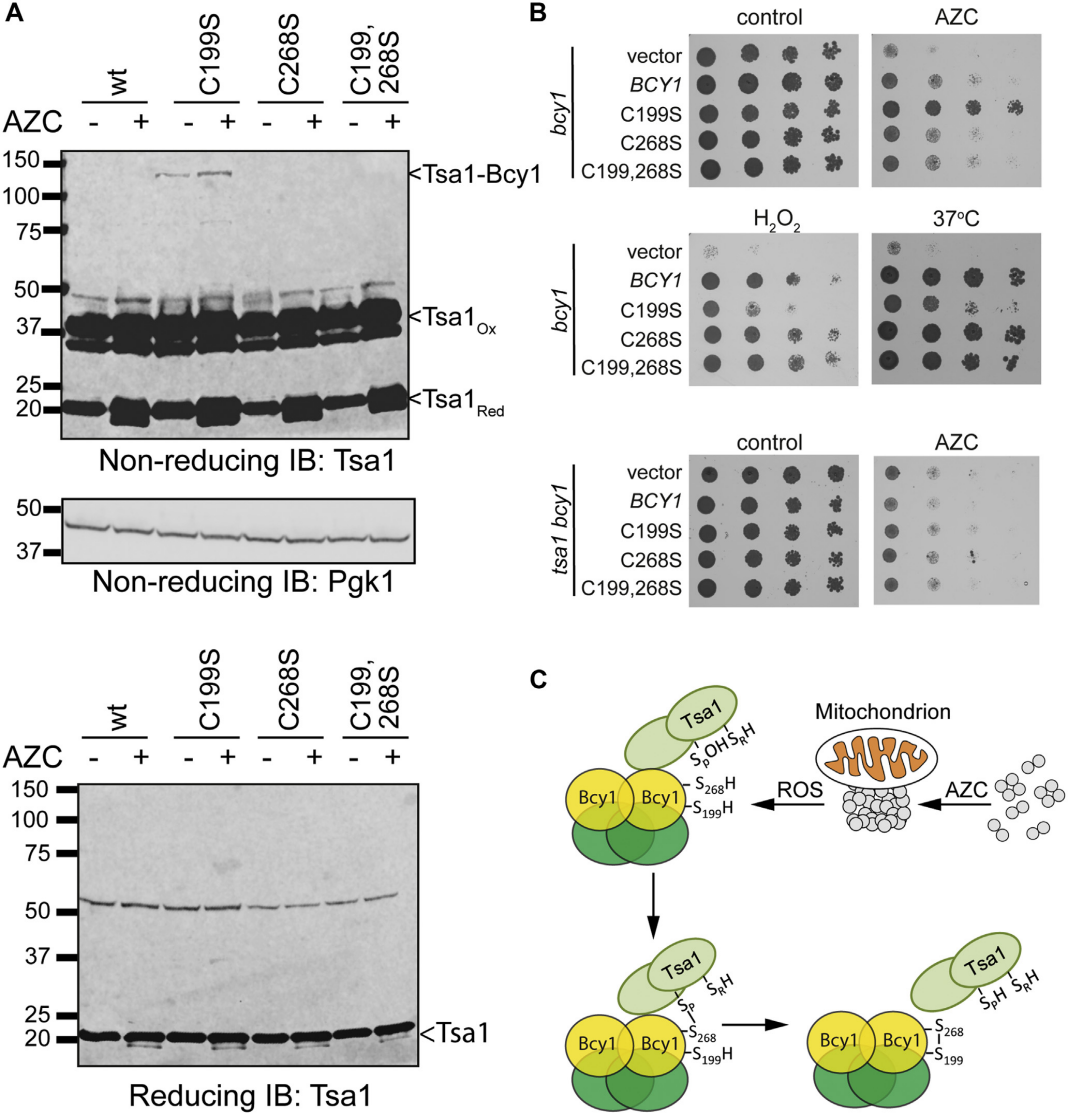
**Mechanism underlying Tsa1-mediated oxidation of Bcy1.***A*, formation of a Tsa1-Bcy1 mixed disulfide conjugate is trapped in the Bcy1 C199S mutant. Proteins from the indicated strains were separated using reducing or nonreducing SDS-PAGE as indicated. Blots were probed with antibodies specific for Tsa1 and Pgk1 is included as a loading control. *B*, mutation of Bcy1 cysteine residues influences stress sensitivity. Results are shown for the *bcy1* mutant containing vector alone (V) or plasmids expressing wild-type *BCY1*, single cysteine (C199S and C268S) and double cysteine (C199, 268S) mutants. Strains were grown to exponential phase and the A_600_ adjusted to 1, 0.1, 0.01, or 0.001 before spotting onto SD plates containing 0.5 mM AZC, 0.6 mM hydrogen peroxide, or incubation at 37 °C. Results are also shown for the *tsa1 bcy1* mutant containing vector alone (V) or plasmids expressing wild-type *BCY1*, single cysteine (C199S and C268S) and double cysteine (C199, 268S) mutants. Strains were grown to exponential phase and the A_600_ adjusted to 1, 0.1, 0.01, or 0.001 before spotting onto SD plates containing 0.3 mM AZC. *C*, model depicting the role of Tsa1 in the signaling response to protein misfolding stress. Tsa1 functions as an antioxidant that detoxifies the localized ROS generated by mitochondrially associated protein aggregates. Tsa1 is found associated with Bcy1 where the Cys268 thiol group in Bcy1 can compete with the thiol group on the Tsa1 resolving cysteine (Cys171) residue for condensation with the sulfenylated peroxidatic cysteine residue (Cys48) of Tsa1. The resulting Tsa1-Bcy1 mixed disulfide then rearranges to yield fully oxidized Bcy1 with a disulfide bond between its two cysteine residues. Following Bcy1 oxidation, Tsa1 is released in its fully reduced form ready to function in subsequent catalytic cycles. Oxidation of Bcy1 reduces cAMP binding and suppresses PKA activity.

Having shown that Tsa1 interacts with Bcy1 in a redox-dependent manner upon protein misfolding stress, we examined the Tsa1-Bcy1 interaction in Bcy1 cysteine mutant strains. We reasoned that any transient mixed disulfide conjugate formed between Tsa1 and Bcy1 might be stabilized by mutating either Cys199 or Cys268 in Bcy1. Strains expressing the single (C199S or C268S) or double (C199, 268S) mutant versions of Bcy1 were left untreated or treated with AZC and whole-cell extracts prepared in the presence of the thiol-blocking agent N-ethylmaleimide (NEM) to prevent further thiol–disulfide exchange reactions and to stabilize any transient mixed disulfides. Immunoblot analysis performed under nonreducing conditions revealed the presence of a Tsa1-Bcy1 mixed disulfide conjugate that was only detected in the C199S mutant (Fig. 6*A*). This Tsa1-Bcy1 mixed disulfide conjugate was resolved under reducing conditions confirming that its formation is redox-dependent (Fig. 6*A*, lower panel). These data suggest that a mixed disulfide is initially formed between the peroxidatic cysteine residue of Tsa1 and Cys268 in Bcy1 and that loss of Cys199 stabilizes the disulfide conjugate (see Discussion for more details). It seems likely therefore that the stabilization of this Tsa1-Bcy1 mixed disulfide conjugate also accounts for the decreased cAMP binding observed in the C199S mutant (Fig. 5*D*).

Having found that a Tsa1-Bcy1 mixed disulfide conjugate is stabilized in the C199S mutant and cAMP binding is reduced in this mutant, we next examined whether the C199S mutant influences resistance to AZC stress. To test AZC sensitivity, we used a plate test assay to assess basal AZC tolerance levels in the different mutant strains. Strains expressing the single C268S or double C199,268S mutant versions of Bcy1 displayed wild-type AZC tolerance (Fig. 6*B*). This correlates with the finding that the C268S and C199,268S mutants bind cAMP at similar levels to wild-type Bcy1 (Fig. 5*D*) and hence would be expected to have similar tolerance to the wild-type. In contrast, the C199S mutant showed increased tolerance to AZC suggesting that stabilizing the Tsa1-Bcy1 mixed disulfide conjugate and the resulting decrease in cAMP binding promote tolerance to protein misfolding stress (Fig. 6*B*). The C199S mutant was more sensitive to hydrogen peroxide stress, reinforcing the idea that cells respond differently to oxidative stresses caused by exogenous hydrogen peroxide *versus* protein misfolding stress (Fig. 6*B*). We also examined the impact of expressing the AZC-resistant Bcy1 C199S mutant in a *tsa1* mutant (Fig. 6*B*). No differences in AZC sensitivity were found in a *tsa1* mutant containing wild-type or Bcy1 cysteine mutant (C199S, C268S, double) versions of Bcy1. This is consistent with a mechanism where stabilization of the Tsa1-Bcy1 mixed disulfide conjugate in the C199S mutant accounts for tolerance to protein misfolding stress (Fig. 6*C*).

## Discussion

Many links have been established between protein aggregation and oxidative stress, although it is often unclear whether ROS promote protein aggregation, or conversely, whether aggregate formation causes ROS generation (45, 46). For example, ROS have frequently been implicated in protein oxidative damage and partially misfolded proteins are more susceptible to oxidation and aggregation (47). Protein aggregation is often accompanied by an increase in oxidative damage to cells and in many aggregation diseases, oxidative stress is an integral part of the pathology (48). Protein misfolding and aggregation can cause the production of mitochondrially derived ROS, which may involve mitochondrial membrane disruption, although the underlying mechanisms remain unclear (13, 45, 49, 50). We have previously shown that the Tsa1 Prx localizes with protein aggregates adjacent to mitochondria, where it is ideally placed to detoxify ROS directly (13). In this current study, we have addressed the role of Tsa1 in mitigating the toxic consequences of protein aggregation and uncovered a novel redox regulatory mechanism that directly controls the cAMP/PKA pathway to respond to protein aggregation and the resulting oxidative stress.

PKA activity has a major impact on gene expression, positively regulating genes associated with rapid growth including metabolism, ribosomal biogenesis, and cell division (37). Conversely, PKA negatively regulates properties associated with slow, respiratory growth and stress responses, including the Msn2/4 transcription factors. Msn2/4 regulate the transcription of many stress response genes that are required for diverse stresses as part of the ESR (26, 27). The activity of the Msn2/4 transcription factors has long been known to be negatively regulated by the cAMP/PKA pathway including *via* phosphorylation mediated by PKA (39, 51, 52, 53). Msn2/4 activity itself is subject to complex control in response to many different stress and growth conditions *via* the integration of diverse control mechanisms (22, 54). Our data indicate that the scope of the transcriptional response to protein misfolding stress is largely similar in wild-type and *tsa1* mutant strains, but the magnitude of the response is dampened in a strain lacking Tsa1. This means that a *tsa1* mutant can still mount an ESR, but many genes are not as upregulated or not as downregulated compared with a wild-type strain, which causes sensitivity to protein misfolding stress.

We identified a direct interaction between the Bcy1 regulatory subunit of PKA and Tsa1 that is present under normal growth conditions. This interaction was increased in a redox-dependent manner mediated *via* Tsa1-dependent oxidation of Bcy1 in response to protein misfolding stress. Mutating the peroxidatic cysteine residue (Cys48) of Tsa1 suppressed the increased Tsa1-Bcy1 interaction observed in response to protein misfolding stress but did not prevent this interaction during normal growth conditions. This indicates that the association of Tsa1 with Bcy1 does not normally require a disulfide bond, but protein aggregate formation promotes an additional redox-dependent interaction between Tsa1 and Bcy1. The increased interaction between Tsa1 and Bcy1 was not significantly increased in response to an oxidative stress directly caused by exogenous hydrogen peroxide addition and no increase in Bcy1 oxidation was detected. This might indicate that localized endogenous ROS generation in response to protein misfolding and aggregation is required to induce Tsa1-mediated Bcy1 oxidation. The aggregates formed upon AZC and H_2_O_2_ exposure are different, since peroxide stress causes the formation of mainly single foci in cells, *versus* the multiple mitochondria-associated foci formed following protein misfolding stress (13, 14). Additionally, ROS generation because of protein aggregate formation is liable to be a slower and more gradual process, compared with the addition of a bolus of hydrogen peroxide. This might have different biological outcomes as illustrated by the overoxidation of Tsa1 that occurs only following hydrogen peroxide addition, but not following nascent protein misfolding stress.

Redox regulation has long been implicated in the control of mammalian PKA enzymes, and, for example, kinase oxidation was originally reported to affect the interaction between its catalytic and regulatory subunits (55, 56, 57). More recently, hydrogen peroxide stress has been found to inhibit *S. cerevisiae* Tpk1 *via* Prx-mediated sulfenylation and oxidation of Ser/Thr protein kinases is emerging as a conserved mechanism that regulates diverse eukaryotic protein kinases (23, 58, 59). However, *S. cerevisiae* has three catalytic subunits (Tpk1–Tpk3) that regulate distinct arms of the PKA pathway and any isoform specificity remains unclear. Oxidation of Bcy1 could therefore provide a mechanism to ensure global downregulation of PKA activity that can affect all three catalytic subunits. There is a precedent for redox regulation of PKA regulatory subunits in mammalian cells, although PKA activity is stimulated rather than inhibited by oxidation, in a mechanism that does not alter cAMP binding (60, 61, 62). Oxidation increases kinase affinity with protein kinase A anchor proteins (AKAPs), which are scaffold proteins responsible for determining the subcellular localization of the holoenzyme. Translocation is thought to bring PKA in closer proximity to its substrates, which sensitizes the kinase to cAMP, allowing the catalytic subunit to phosphorylate substrate proteins. Structural and sequence analysis of yeast Bcy1 revealed that the N-terminal region responsible for dimerization and for docking to AKAPs (D/D domain) in higher eukaryotic R1 subunits is not well conserved in yeast (63). Bcy1 also lacks the two redox sensor cysteine residues that are oxidized by hydrogen peroxide in mammalian cells and regulation *via* AKAP interaction remains ill-defined in fungi (64).

In contrast to mammalian R1 proteins, Bcy1 only contains two cysteine residues, and these are both localized within the region of Bcy1 where cAMP binding occurs at its C-terminus. We found that Tsa1 promotes the formation of a disulfide bond in Bcy1 that acts to suppress cAMP binding, which in turn reduces PKA activity to promote tolerance to protein misfolding stress. The proposed mechanism shown in Figure 6*C* highlights the role of Tsa1 as an antioxidant that normally acts to detoxify the localized ROS generated by mitochondrially associated protein aggregates (13). The association of Tsa1 with Bcy1 means that the thiol group on Cys268 in Bcy1 can compete with the thiol group on the Tsa1 resolving cysteine (Cys171) residue for condensation with the sulfenylated peroxidatic cysteine residue (Cys48) of Tsa1. The resulting mixed disulfide then rearranges to yield fully oxidized Bcy1 with a disulfide bond formed between its two cysteine residues. Following Bcy1 oxidation, Tsa1 will be in its fully reduced form ready to function in subsequent catalytic cycles. Oxidized Bcy1 may ultimately be reduced by a thioredoxin or other oxidoreductase, similar to other peroxidase-based redox relay systems.

Our current data highlight a new signaling role for Tsa1 in the localized control of the PKA pathway that is required for the response to protein misfolding stress. Oxidation of the regulatory subunit of PKA enables additional fine-tuning of signaling mediated *via* the cAMP second messenger, adding another level of control to mediate PKA activity. Directly controlling the regulatory subunit in response to protein misfolding stress provides a mechanism to globally moderate cellular PKA holoenzyme activity, independent of any isoform specificity. Redox regulation of the PKA regulatory subunit provides a previously unrecognized mechanism for protein oxidation to signal protein phosphorylation, linking these two major forms of posttranslational modification.

## Experimental procedures

### Yeast strains and plasmids

*S. cerevisiae* strains used in this study were isogenic derivatives of W303 (*MAT*a *ura3-52 leu2-3 leu2-112 trp1-1 ade2-1 his3-11 can1-100*). Strains deleted for *TSA1* (*tsa1::URA3 or tsa1::LEU2*), *BCY1* (*bcy1::URA3*) and *MSN2 MSN4* (*msn2::HIS3 msn4::TRP1*) have all been described previously (10, 65, 66). The three *tpk*^*wk*^ mutants, *tpk1*^*wk*^ (MATa *ade8 his3 leu2 trp1 ura3 tpk1*^*w1*^ *tpk2∷HIS3 tpk3∷TRP1*), *tpk2*^*wk*^ (MATa *ade8 his3 leu2 trp1 ura3 tpk1∷URA3 tpk2*^*w1*^ *tpk3∷TRP1*), and *tpk3*^*wk*^ (MATa *ade8 his3 leu2 trp1 ura3 tpk1∷URA3 tpk2∷HIS3 tpk3*^*w1*^) are isogenic derivatives of SP1 (*MATa ade8 his3 leu2 trp1 ura3*) (41). Chromosomal *BCY1* was epitope tagged with nine Myc epitopes using a PCR-based strategy with plasmid pYM6 (67). Strain W303 containing Myc-tagged Bcy1 was deleted for *TPK1* (*tpk1::*KanMX) and strain BY4741 containing TAP-tagged Maf1 (*MATa his3Δ1 leu2Δ0 met15Δ0 ura3Δ0 MAF1-TAP::HIS3*) was deleted for *TSA1* (t*sa1::LEU2*) using standard yeast methodology.

Plasmids expressing wild-type *TSA1* or single (C48S, C171S) and double (C48,171S) mutants have been described previously (68, 69, 70). Plasmid pRS316-Myc-Tsa1 is described in (71) and the C171S mutant version was created using site-directed mutagenesis. Plasmids expressing FLAG-tagged wild-type Bcy1, single cysteine mutants (C199S, C268S) or double cysteine mutant versions of Bcy1 (C199, 268S) were constructed by cloning commercially synthesized gene fragments into plasmid pRS413 (72).

### Growth and stress conditions

Yeast strains were grown in minimal SD medium (0.17% w/v yeast nitrogen base without amino acids with 0.5% w/v ammonium sulfate, 2% w/v glucose supplemented with appropriate amino acids and bases). Media were solidified by the addition of 2% (w/v) agar. For stress conditions in liquid cultures, hydrogen peroxide (0.4 mM) or AZC (5 mM) was added for 2 h. For lower H_2_O_2_ titration experiments, 0.1 or 0.4 mM H_2_O_2_ was added for 10 or 30 min. For the analysis of growth on plates, cultures were grown until mid-exponential phase, serially diluted (A_600_ = 1.0, 0.1, 0.01 and 0.001), and spotted onto agar plates containing the indicated concentrations of hydrogen peroxide or AZC. Heat stress was induced by incubating plates at the indicated temperatures.

### RNA-seq analysis

Total RNA was isolated using Trizol (Life Technologies) and quantified using a Nanodrop 8000 spectrophotometer (Thermo Fisher Scientific). rRNA was depleted using the Ribominus Eukaryote Kit for RNA-Seq (Life Technologies). Sequencing reads were mapped to the *S. cerevisiae* genome (http://downloads.yeastgenome.org/sequence/S288C_reference/chromosomes/) using the STAR RNA-seq aligner (73). Variability between the samples in the levels of rRNA depletion, rRNA and tRNA mapping reads was removed using RseQC (74). Cufflinks package v.2.2.1 was used for subsequent quantification of gene expression and differential expression testing (75).

Hsf1 and Msn2/4 targets are defined in (76) including 43 known targets of Hsf1 and 231 known targets of Mns2/4. These are mainly different transcripts since the overlap is quite small (six transcripts are targets of both Hsf1 and Msn2/4). Ribosomal proteins are defined in (76) and Yap1 targets were defined using (77). Enrichment for transcription factor binding sites was identified using oPOSSUM (77). The transcriptional response to AZC stress was compared with heat stress (25) or oxidative stress induced by diamide (76).

### Immunoprecipitation and protein identification experiments

A *tsa1* deletion mutant containing Myc-Tsa1-C171S was grown to mid-exponential phase in SD media and treated with hydrogen peroxide or AZC for 2 h. A control *tsa1* mutant strain containing vector alone was included for comparison. Cells were lysed in lysis buffer (100 mM PBS pH 7.4, 1 mM PMSF, containing a protease inhibitors cocktail, Roche). Protein extracts were incubated with EZ red anti-myc affinity beads (Sigma), pre-equilibrated in IPP50 buffer (10 mM Tris pH 8.0, 50 mM NaCl), for 4 h at 4 °C. Beads were washed three times with IPP50 buffer and proteins eluted using 2x BOLT Sample buffer (Thermo Scientific) at 95 °C. For protein identification, protein samples were run a short distance into SDS-PAGE gels and stained using colloidal Coomassie blue (Sigma). Total proteins were excised, trypsin digested and identified using liquid chromatography–mass spectrometry (performed by the Biomolecular Analysis Core Facility, The University of Manchester) as described below. Proteins that were identified in two out of three repeats of the empty vector control were considered unspecific and proteins were considered specific if they were identified in two out three biological repeats.

To verify the interaction between Tsa1 and Bcy1, Bcy1-Myc was immunoprecipitated using anti-c-Myc magnetic beads (Thermo Scientific) from a *tsa1* deletion strain complemented with plasmids expressing wild-type or mutant untagged versions of Tsa1. Cells were lysed in breakage buffer (25 mM sodium buffer pH 7.5, 50 mM *N*-ethylmaleimide, 1 mM PMSF, containing a protease inhibitors cocktail, Roche). Protein extracts were incubated with anti-c-Myc magnetic beads, pre-equilibrated in wash buffer (500 mM NaCl, 20 mM Tris-HCl pH 8.0, 2 mM MgCl_2_, 0.5% nonident P40). Beads were washed three times with wash buffer and bound proteins eluted at 95 °C. For the analysis of Maf1 phosphorylation, cells were lysed in breakage buffer (20 mM Tris pH 8, 100 mM NaCl, 1 mM MgCl_2_, 0.1% (v/v) NP40 containing a protease inhibitors cocktail, Roche and PhosStop, Sigma). Protein extracts were incubated with Pan Mouse IgG Dynabeads (Invitrogen), pre-equilibrated in wash buffer (20 mM Tris pH 8, 100 mM NaCl, 1 mM MgCl_2_, 0.5% (v/v) NP40). Beads were washed three times with wash buffer and bound proteins eluted at 95 °C.

### Mass spectrometry sample and data processing

Proteins were identified using a Thermo Scientific Velos Pro Mass Spectrometer. The digestion enzyme was Trypsin (cleaves on R and K except for C-terminal P obstructing it; not independent; not semi-specific, *i.e.*, fully specific). The Max # accepted missed cleavages: 1; Fixed modifications: Carbamidomethyl (C) (+57), Variable modifications: Oxidation (M) (+16); Precursor tolerance: 0.6 Da; Fragmentation tolerance: 0.5 Da. The software generating the peaklist was Thermo’s ExtractMSN (version 5.0) and the search engine was Mascot v2.4.1. The database searched was SGD (version 2015-01-13; 6713 entries (or proteins) searched). The estimation of false discovery rate (FDR) for the protein level was 0.1% with an FDR of 8.7% to 10.3% at the peptide level calculated using Protein Prophet and Peptide Prophet. The version of Scaffold used to generate the report was 4.11.0. Processed data are shown in Table S1. The mass spectrometry proteomics data have been deposited to the ProteomeXchange Consortium *via* the PRIDE (78) partner repository with the dataset identifier PXD023668.

### Protein and redox state analysis

The redox state of Tsa1 or Bcy1 was assessed as described previously (79) except the thiol-reactive probe 4-acetamido-4’maleimidyldystilbene-2,2’ disulfonic acid (AMS) was substituted for methoxypolyethylene glycol maleimide ((mPEG-MAL)-5000, Sigma). Protein extracts were electrophoresed on reducing NuPAGE protein minigels (Life Technologies) and electroblotted onto nitrocellulose membranes. Bound antibody was visualized using WesternSure Chemiluminescent Reagents (LI-COR). For nonreducing western blot analysis, proteins were extracted in the presence of N-ethylmaleimide (NEM) to prevent any nonspecific thiol oxidation during sample preparation. Primary antibodies used were Tsa1 (70), FLAG (F3165, Sigma), Myc 4A6 (05-724, Millipore), Prx-SO_2/3_ (ab16830, Abcam), phospho-PKA (100G7E, Cell Signaling Technology), and Pgk1 (sc-130335, Santa Cruz).

### cAMP-affinity chromatography

cAMP-agarose purification was based on a previously described protocol (64). Briefly, cells were harvested by centrifugation at 4000 rpm, 4 °C, for 5 min. Cells were broken with glass beads in lysis buffer (50 mM sodium phosphate, 150 mM Nacl, 2 mM EDTA, 0.1% (v/v) NP-40, pH 7.4; plus protease inhibitors) using a Minibead beater (Biospec Scientific, Bartlesville) for 45 s at 4 °C The supernatant was centrifuged at 13,000*g* for 5 min at 4 °C and 75 μg of protein extract incubated over-night at 4 °C with 50 μl of adenosine 3,5-cyclic monophosphate-agarose (Sigma). The resin was washed three times with lysis buffer and bound protein eluted by heating at 95 °C for 5 min. The specificity of binding was confirmed by including 50 mM cAMP in the incubation step and showing that it prevented binding of Bcy1 to the resin.

## Data availability

Sequencing data are publicly available on ArrayExpress, E-MTAB-9355. Mass spectrometry data are available *via* ProteomeXchange with identifier PXD023668.

## Supporting information

This article contains supporting information (25, 76).

## Conflict of interest

The authors declare that they have no conflicts of interest with the contents of this article.
