# Supplementary material for: Tolerance to nascent protein misfolding stress requires fine-tuning of the cAMP/PKA pathway
Source: J Biol Chem. 2021 Apr 22;296:100690. doi: 10.1016/j.jbc.2021.100690 (PMC8164027; doi:10.1016/j.jbc.2021.100690)
Supplement: Supplemental Figures S1 and S2 [file mmc2.pdf]

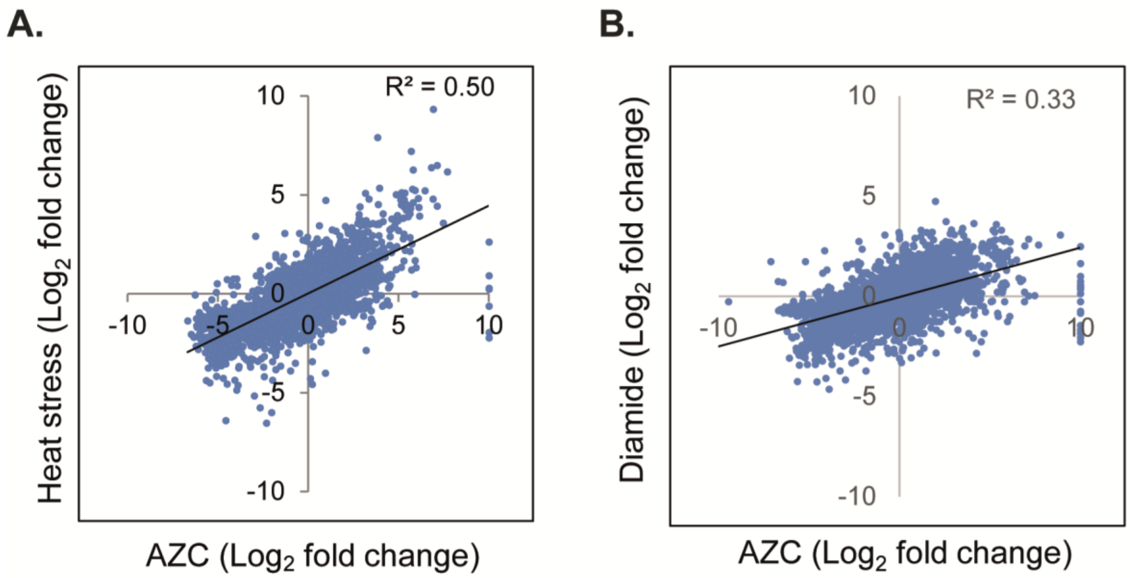

Supplementary Fig. 1. The transcriptional response to protein misfolding stress is more similar to heat stress than oxidative stress. Scatterplots are shown comparing the transcriptional changes in response to AZC stress with heat stress (25) (**A)** or oxidative stress caused by diamide (76) **(B)**.

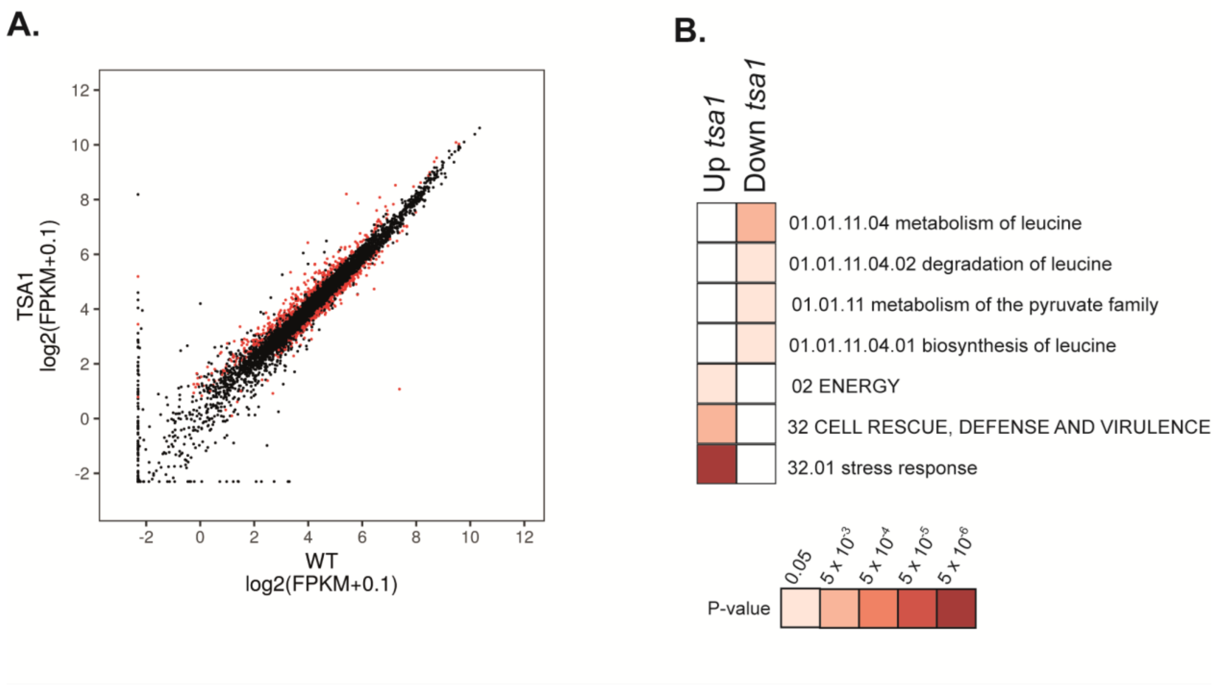

Supplementary Fig. 2. Modest changes in gene expression are observed in a *tsa1* mutant in the absence of stress. A. 170 and 34 transcripts are up- or downregulated by greater than two-fold in a *tsa1* compared with a wild type strain, respectively. A scatterplot is shown comparing the transcriptional changes in response to AZC for each strain. **B.** Significantly enriched functional categories within these transcripts were determined (FDR < 5%) and results are ordered on MIPS category classification. The downregulated transcripts were enriched for functions related to leucine metabolism reflecting the fact that *TSA1* is deleted with the *LEU2* marker highlighting the sensitivity of the RNA-Seq experiment. Confidence of each classification category is shown as Bonferroni corrected *p*-values.
